# Standard Operating Procedure for the determination of trace elements in hydrothermal fluids by Inductively Coupled Plasma Mass Spectrometry (ICP-MS)

**DOI:** 10.12688/openreseurope.15699.2

**Published:** 2024-07-24

**Authors:** Monica Correggia, Luciano Di Iorio, Alessia Benedicta Bastianoni, Mustafa Yücel, Angelina Cordone, Donato Giovannelli

**Affiliations:** 1 Department of Biology, Universita degli Studi di Napoli Federico II, Naples, Campania, Italy; 2 Institute of Marine Sciences, Orta Dogu Teknik Universitesi, Ankara, Ankara, Turkey; 3 Earth-Life Science Institute, ELSI, Tokyo Kogyo Daigaku, Meguro, Tokyo, Japan; 4 Department of Marine and Coastal Science, Rutgers University New Brunswick, New Brunswick, New Jersey, USA; 5 Istituto per le Risorse Biologiche e Biotecnologiche Marine, Consiglio Nazionale delle Ricerche, CNR-IRBIM, Ancona, Italy; 6 Marine Chemistry & Geochemistry Department, Woods Hole Oceanographic Institution, Woods Hole, Massachusetts, USA

**Keywords:** trace element, hydrothermal fluids, inductively coupled plasma mass spectrometry, Standard Operating Procedure, aqueous geochemistry

## Abstract

This standard operating procedure (SOP) validates an inductively coupled plasma mass spectrometry (ICP-MS) procedure for the determination of trace elements in hydrothermal fluids. Hydrothermal fluids are aqueous solutions with a wide range of temperature, salinity, pH and trace elements that can be used by a set of microbial proteins containing redox-sensitive transition metals as their catalytic core. Due to the high variability of these samples, we have developed this protocol taking into account the special features of the matrices analyzed. An ICP-MS 7900 Agilent system was used. Calibration curves are linear in the 0.01 to 100 μg/L concentration range.

## Plain language summary

This manuscript outlines the standard operating procedure used to determine trace elements in hydrothermal fluids, which are characterized by a high variability of physical-chemical parameters. Due to the high variability of our samples, we customized the method to compensate for the variability on the physico-chemical parameters. The obtained data on the distribution and abundance of trace elements in sediments and fluids can be used to track geochemical processes mobilizing the metals as well as their influence on the microbial diversity in geothermal systems.

## Introduction

This Standard Operating Procedure (SOP) was developed in the Giovannelli Lab at the Department of Biology of the University of Naples Federico II and outlines the determination of trace elements involved in biogeochemical cycles from solid and liquid matrices using inductively coupled plasma mass spectrometry (ICP-MS). Although trace metals are recognized as important for microbial metabolism, they are not classically considered as the main factors that drive functional microbial diversity ( Giovannelli, 2023a). Generally, microbial diversity is linked toa number of physical and chemical parameters including, but not limited to, temperature, pH, pressure, salinity, O _2_ availability or substrates ( Delgado-Baquerizo *et al.*, 2018). The role of metals in influencing biodiversity has been studied classically in the context of their toxicity. By contrast, information on minimum concentration requirements for specific trace elements is severely limited and very few studies have investigated their role in microbial functional diversity control ( Bertrand *et al.*, 2007; Giovannelli, 2023a; Morel & Price, 2003). Microorganisms play a crucial role in biogeochemical cycles as they are involved in redox reaction, processes that clearly alter the composition of biosphere and geosphere, which have evolved over time, influencing each other. Biogeochemical cycles play a key role in controlling the interaction between geosphere and biosphere ( Falkowski *et al.*, 2008; Jelen *et al.*, 2016; Moore *et al.*, 2017). Trace metals such as Fe, Co, Ni, Mo, W, V and Cu are used in a set of microbial proteins containing redox-sensitive transition metals as their core catalytic ( Hay Mele *et al.*, 2023) but, despite the importance of this process, this relation has not been investigated in detail. Moreover, the availability of transition metal and substrates has changed over the course of Earth’s history as a result of changing redox conditions, particularly global oxygenation ( Anbar, 2008). This evolution has allowed microbes to access a larger number of redox couples ( Jelen *et al.*, 2016). Thus, an additional level of explanatory power could be provided by the availability of metals required for the key enzymes required for the different variants of each pathway, as demonstrated by recent work in convergent margins ( Fullerton *et al.*, 2021; Rogers *et al.*, 2022).

Deeply-sourced seeps, encompassing a wide variety of diverse secondary geothermal features ( Giovannelli *et al.*, 2022), have a wide range of temperature, pH and salinity. Our dataset routinely includes samples with pH varying between 0.5 to 11.2, temperatures ranging from 2 °C to 375 °C and salinity from 0 % to 35 %. Traditional trace elements determination of geothermal waters focuses on elements that are used to understand geochemical processes mainly linked to the source of the fluids ( Féraud *et al.*, 2009), the extent of water-rock interactions ( Grandell *et al.*, 2016) or the presence of anomalies of economically valuable metals also in the future market of clean energy technology such as gold, lithium, cobalt ( Gianelli & Grassi, 2001). Comparatively less attention has been devoted to the biologically relevant elements ( Giovannelli, 2023a), and very often datasets lack information on rock composition, fluid geochemistry involving the key biological elements such as molybdenum, vanadium or cobalt. Here we describe an SOP developed to routinely analyze such a wide range of fluid and sediments obtained from deeply-sourced springs. The described SOP aims at measuring a wide range of elements, including the classically determined elements of geological interest as well as the complete set of biological metals.

## Methods

ICP-MS is a technique that combines mass spectrometry to inductively coupled plasma, useful for determining trace metallic and non-metallic inorganic substances present in a sample. Liquid samples are taken through a peristaltic pump and nebulized, and the resulting aerosol is transported to the plasma torch (generally based on Argon gas) where the elements are ionized. The ions pass through a vacuum chamber where they are separated into photons, neutral particles and interfering ions by an ion lens and a collision chamber (based on diverse inert or reactive gasses depending on the final application). Analytes arrive at the quadrupole mass filter and are kept in a state of vacuum to minimize interferences. Finally, each ion strikes through the detector to be counted. In a few minutes ICP-MS is capable of giving quantitative information on a large swath of elements with theoretical limits of detection in the range of 1 ppt to 10 ppb depending on the specific element ( Thomas, 2003).

We have developed this protocol using an Agilent ICP-MS 7900 (G8403A). This SOP, with minimal instrument specific modifications, can be carried out using other similar instruments. As previously mentioned, hydrothermal fluids are aqueous solutions with a wide range of temperature, salinity and pH values. So, compared to the routine analysis of drinking water or seawater we have developed specific adjustments because the biggest problem during this type of analysis is the amount of suspended particulate matter and the salinity. Some matrix components may deposit around the sampling and the skimmer cones and can lead to long-term signal instability and potentially nebuliser blockage. For example, matrices high in NaCl can form volatile oxides that can settle on cones and be released later on during the run, compromising subsequent samples. As hydrothermal fluids and hydrothermal sediment digestates tend to have high salinities (>3 % and up to 30 %), care must be taken in diluting the sample while considering the effects of dilution on the detection limits of each element. For comparison, it was reported that with a 50 % dilution of seawater (1.5 % NaCl final concentrations) there is no significant cone blockage and signal stability remains good ( Thomas, 2003).

A common problem in ICP-MS analysis is the possible formation of interferences ( Olesik, 2014), like polyatomic interference that occur when two or more elements combine and have the same mass as an element of interest (for example ^35^Cl ^16^O ^+^ an ^51^V ^+^), fortunately most of the spectral overlaps can be reduced by the collision cell.

Finally, there are many factors that can affect the final trace metal determination results. Given the high sensitivity of the instrument (up to a few ppt for most metals), great care must be taken to minimize external contamination to the used equipment. To minimize possible contamination during field sampling and laboratory preparation procedures we always use specifically selected materials that have been treated to reduce the amount of contaminating metals (see below for details). For example, non-colored plastic tips and vials are preferred to colored ones, as the dye used for coloring the plastic can leach elements such as Cu, Fe, Zn and Cd, and all plastic and glass vials are acid washed to prevent leaching of Sb, Zn, Mn, Fe, Ba ( Moody, 1983; Thomas, 2003). Indeed, it is preferable not to keep the samples in glass vials for a long period especially if the concentration of these elements are extremely low. The ICP-MS laboratory has restricted access to external personnel, and specific personal protective devices are always used to minimize common contamination from skin, hair, nails or jewelry.

### Principle of functioning of ICP-MS

The sample is placed in the autosampler, and a peristaltic pump transports it to the nebulizer, where the liquid sample is converted into aerosol using argon gas. The aerosol passes through a spray chamber, where the larger droplets are removed. The fine droplets are carried by the argon gas flow to the ICP plasma torch. The aerosol droplets are carried through the center of the plasma, where the droplets are dried, decomposed, dissociated, atomized and finally ionized. The sample passes through a vacuum interface, interface cones that provide optimum vacuum conditions for operation of the quadrupole mass filter and detector, and ion lens with the aim of separating ions from neutral particles and photons. The ions pass through a collision cell to resolve the spectral overlaps caused by the unwanted ions (polyatomic), which appear at the same mass as the ions of the analyte being measured.

The cell is pressurized with helium (He), a non-reactive gas. The ions collide with the atoms of He, and larger ions such as polyatomic ones are preferentially removed. The ions arrive at the quadrupole mass spectrometer to filter the ions according to the mass-charge ratio (m/z). The mass spectrometer consists of two bar pairs to which an electric field is applied. Alternating electric fields destabilize the trajectories of all ions above and below the set mass, then ions at any mass other than the set mass are repelled by the ion beam. At the end, ions arrive at the electron multiplier that uses a high voltage electrode positioned so that ions that emerge from the quadrupole strike the dynode. For each mass measured, the counts registered by the detector are processed by the data analysis software. For quantitative analysis, the signal measured by the detector is in units of counts per second (CPS) that corresponds to the number of ions striking the detector every second ( Wilschefski & Baxter, 2019). ICP-MS operational conditions of this SOP are described in Table 1.

**Table 1.** ICP-MS operation conditions.

RF	1.5 KW
Plasma Gas	15 L/min
Auxiliary Gas	0.9 L/min
Nebulizer Gas	1.09 L/min
He Flow	4.7 L/min
Spray Chamber Temperature	2 °C
Replicate	3
Nebulizer pump	0.1 rps

### Standard operating practice in the giovannelli lab

**Figure 1.**
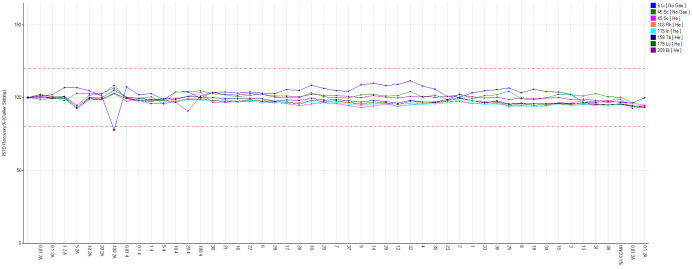
Internal standard recovery during an analytical run.

The method was developed using inductively coupled plasma mass spectrometry (ICP-MS 7900, G8403A). In order to reduce the suspended particulate, all samples are filtered in the field through a 0.22 μm filter, this has the benefit of removing all the particles that might dissolve after acidification and removing all the microbial cells. All plastic and glassware materials used during analysis are washed in an acid bath (see below). Using HNO _3_ as a solvent for the dilutions is preferred to water because some elements are unstable and can co-precipitate. Prior to analysis, sediment samples are digested using microwave assisted digestions following the EPA3051A method and filtered using laboratory filter paper (pre-treated with a 1:5 HNO _3_: H _2_O solution). A certified reference material is used to verify the digestion efficiency. Data acquisition and analysis is carried out through MassHunter 4.6 software provided with the instrument. To convert data into a concentration, calibration standards containing known concentrations of elements are used to construct a calibration curve. The mass and the ionization potential, which may be determinants of the matrix effects, are evaluated through an internal standard at the final concentration of 400 μg/L ( Figure 1). Internal standard is used to correct for changes in instrument operating conditions and sample-specific matrix effects. The same quantity of internal standard is added to each sample, standard and blank, and results are calculated using the ratio of the analyte and internal standard signal. Calibration curves for the trace element of interest are run in the range of 0.01 and 100 ppb using certified multistandard with R ≥ 0.999. Calibration is carried out every time the device is switched on and a 10 ppb multistandard is run every 10 samples to check for recovery. Finally, our system is stabilized and connected to an UPS (MISSION 10000) to avoid switching off the instrument in case of power surges.

### Apparatus and equipment

- 7900 ICP-MS (G8403A Agilent Technologies, USA)
- SPS4 Autosampler (AU204810646, Agilent Technologies, USA)
- ICP-MS MassHunter 4.6 software Version C.01.06 (Agilent Technologies, USA) (free alternative, OpenChrom)
- Multiwave GO Plus, Microwave Digestion System (Anton Paar, Austria)
- Analytical balance capable of 0.0001 g sensitivity (MS105DU Mettler Toledo, USA)
- Graduated Cylinder
- Acid Dispenser
- Pipettes for reagent and standard preparation
- Plastic syringes
- PP syringe filters 0.22 μm (TS-900-063 Perlabo)
- Laboratory filter paper
- PTFE spatula

### Reagents and standards

- Deionized water to wash the plastic material
- Type I water from ultrapure water system with a resistivity of 18 MΩ used for sample and standard preparation (PURIST Ultrapure Water System, Rephile)
- Nitric Acid 67 %, NORMATOM® for trace analysis
- Argon gas high purity (99.999 %)
- Helium gas high purity (99.999 %)
- Hydrochloric Acid 37 % (Reag. USP) for analysis, ACS, ISO
- Stock Multi-element calibration standard-2A, 10 μg/mL of Ag, AI, As, Ba, Be, Ca, Cd, Co, Cr, Cs, Cu, Fe, Ga, K, Li, Mg, Mn, Na, Ni, Pb, Rb, Se, Sr, TI, U, V, Zn; matrix 5 % HNO (8500-6940, Agilent Technologies)
- Stock Multi-element calibration standard-4, 10 μg/mL of B, Ge, Mo, Nb, P, Re, S, Si, Ta, TI, W, Zr; matrix H _2_O/0.2 % HF/trace HNO _3_ (8500-6942, Agilent Technologies)
- Stock Internal standard mix, 100 mg/L of Bi, Ge, In, Li ^6^, Lu, Rh, Sc, Tb; matrix 10 % HNO _3_ (5188-6525, Agilent Technologies)
- Tuning Solution, 1 μg/L Ce, Co, Li, Mg, TI, Y; matrix 2 wt% HNO _3._This multi element solution is used to check the sensitivity and mass resolution of the instrument (5185-5959, Agilent Technologies)
- ENVIR-META-12 interlaboratory test (UNICHIM)

### Cleaning procedure

ICP-MS detects elements even in ppt (parts per trillion), so contamination is a very serious issue. The use of glassware is not recommended due to impurities leaching from the glass. Plastic is usually better than glass, however even these materials can contain leachable contaminants, such as phosphorus or barium compounds, so a cleaning procedure must be carried out to minimize possible contamination.

All material used for the analysis has been subjected to the following treatment to remove leachable contaminants:

- Rinse tips, tubes and caps with deionized water and soap, let it soak for 24 h. This step is used to remove possible organic contamination.
- Wash with deionized water and place in a 0.5 N hydrochloric acid bath, making sure that each material is fully submerged, soak for 12 h. This step is used to remove the possible trace metal contamination.
- Wash with type I water (18 MΩ/cm) five times and shake off excess water. Dry under a chemical hood.

### Sample collection, preservation and storage

In the field, samples are collected and filtered through a 0.22 μm membrane filter in metal free plastic tubes and acidified to HNO _3_ 2 %. Instead, solid samples are collected with a PRFE spatula in a metal free tube. If liquid samples are not processed immediately, they can be stored at 4 °C, solid samples are stored at -20 °C. Store a Nitric acid stock to evaluate the possible metal contamination.

### Sample preparation

***Liquid samples***

1. Gravimetric dilutions are performed sequentially (1:10, 1:100, 1:1,000, 1:10,000) based on the salinity of the samples, using an analytical balance, in acid washed 15 mL falcon tubes with 1 % HNO _3_, max 2 h before the analysis start.
2. Prepare blanks with the same acid used to prepare the dilution.
3. After the analysis, store the samples along with the blank and an aliquot of nitric acid used in the analysis for future references.

***Solid samples***

Before running ICP-MS, samples are overnight dried and microwaved digested following the EPA 3051A method for digestion of sediments, sludges, soils and oil. This digestion provides the total trace metal concentration present in the sample that could be available for microbes.

**Microwave digestion of sediment samples**

1. Dry the homogenized sample, about 2 g, at 60 °C for 48 h (samples should have been stored and taken using metal-free containers).
2. After, weigh about 0.5 g using the analytical balance in a metal free container.
3. Transfer the sample into a clean PTFE digestion vessel and add 12 mL of aqua regia with concentrated HNO _3_ and HCl (3:1). Do this under the chemical hood.
4. Prepare a blank with just 12 mL of aqua regia.
5. Close the vessel and place them inside the rotor considering the recommendations of the manufacturer.
6. Set up the digestion method by selecting “EPA3051A”.
7. Lower the chemical hood glass when the digestion system starts the cooling stage and do not open it until it finishes.
8. Once the digestion is finished and the vessels are cooled (approx. 25–30 min), open the digester and the vessels under the chemical hood. Prepare as many 50 mL volumetric flasks (previously rinsed with 1 % HNO _3_ for 24 h) as samples to be digested and filter them using laboratory filter paper (pre-treated with the 1:5 HNO _3_:H _2_O mixture) placed in a long stem funnel.
9. Bring samples to volume with ultrapure water and store the digestates in metal-free falcon tubes.
10. Use the solution obtained for the preparation of the dilutions to be run with ICP-MS.

Digestion efficiency is verified using ENVIR-META-12 certified test material. Digestion is performed in triplicates; data are expressed as mean ± SD. Our recovery for As, Cd, Co, Cr, Cu, Ni, Pb, V, Tl from ENV-META-12 certified test material (UNICHIM), used for the analysis of different types of matrices such as soil, sediment, sludge and waste is reported in Table 2.

**Table 2.** Results of the analysis of the microwave assessed acid digested reference materials.

Analyte	%Recovery	mg/L ± SD
^75^As	95.9	24.46 ± 0.40
^111^Cd	92.7	2.55 ± 0.04
^59^Co	97	13.1 ± 0.32
^52^Cr	75.2	115.8 ± 1.09
^63^Cu	97.6	89.22 ± 1.72
^60^Ni	98.6	77.09 ± 1.60
^208^Pb	91.6	181.36 ± 2.15
^51^V	75.2	63.21 ± 0.13
^205^Tl	93.5	3.92 ± 0.04
^66^Zn	90.7	241.27 ± 1.45
^9^Be	93.8	1.21 ± 0.03

### Quality control (QC) of the analytical setup

**Figure 2.**
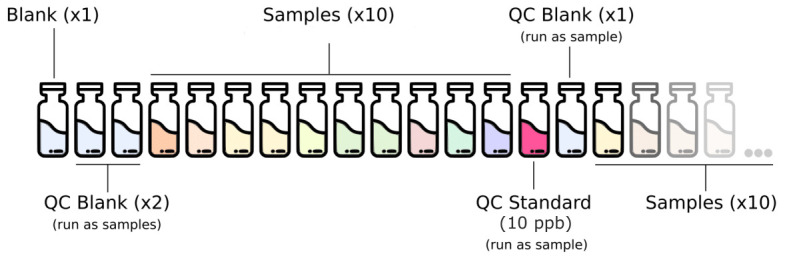
Analytical setup for the routine analysis of trace metal in deeply-sourced seeps fluid samples.

The full analytical setup consists of a series of quality control (QC) blanks, samples and QC standard run in a specific order described in Figure 2. The QC blanks are used to verify the absence of elements carryover and confirm low background levels while the QC standard is used to check for peak drift and consistent quantification of the standards.

QC, quality control.

- Calibration is performed every time the device is switched on and repeated when the QC step fails while using the routine standard 10 ppb.
- Blanks composed of 1 % HNO _3_ are run at the beginning of every run.
- Two QC blanks 1 % HNO _3_ are run as samples at the beginning of each run and once every 10 samples after the routine QC standard.
- The 10 ppb calibration standard solution is run as a sample for routine QC verification once every 10 samples.

### Creation of the method

- Switch on exhaust fume and peristaltic pump;
- Open the gas valve;
- Turn on the Autosampler and Chiller;
- Turn on the Plasma and wait about 40 min for the tuning and the instrument set up;
- Create the Batch and choose the Methods. Usually, we use the “General Purpose method” (high sensitivity) for typical aqueous or acid digested samples (< 0.1 % TDS). Instead, for aqueous or acid digested samples with high TDS content where exceptionally high matrix tolerance is required use the “High Matrix method” (low sensitivity) in the 50 to 300 μg/L standard concentration range. In “Acquisition Parameters” add or remove the element and choose the gas mode (He or No Gas). In “Acquisition methods” add the standard concentration and levels. In “Sample list” make a sample list analysis with relative information like expedition, sample origin, sample type, position and dilution applied to the sample;
- Perform the Autotune step to check the instrument sensitivity;
- Validate the Methods and add to “Queue”.

### Instrument calibration

**Figure 3.**
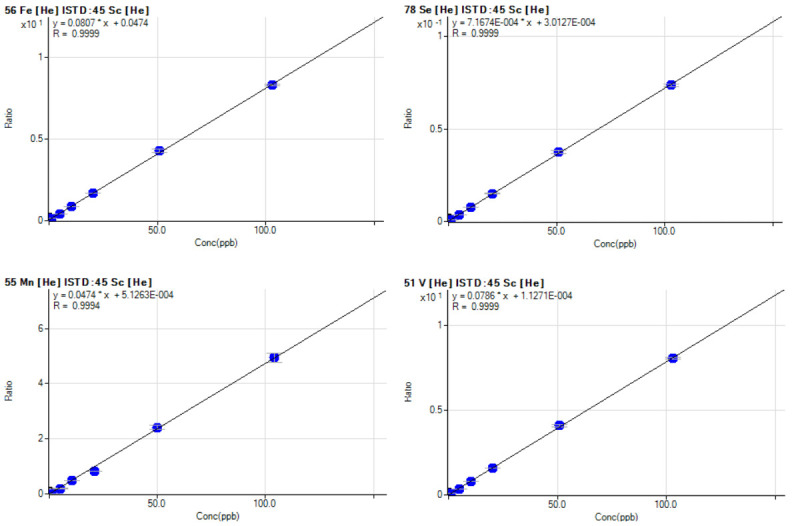
Example of calibration curves for Fe, Se, Mn and V with a final concentration of 0.01, 0.1, 1, 5, 10, 20, 50, 100 μg/L determined following the described SOP.

In our laboratory we routinely quantify biometal such as V, Mn, Fe, Co, Ni, Cu, As, Se, Mo, Cd and W ( Giovannelli, 2022), and other elements such as Ti, Cr, Ga, Rb, Sr, Zr, Nb, Ag, Cs, Ba, Ta, Re, Tl, Pb, U. Moreover, in the next future we will also quantify rare earth elements such as Ce, Dy, Er, Eu, Gd, Ho, La, Lu, Nd, Pr, Sm, Th, Tm, Y, Yb. The signal measured by ICP-MS is in count per second, in order to convert this into a concentration value, external calibration standards containing known concentration of elements are used to produce a calibration curve. Calibration is carried out every time the device is switched on and in case of failed QC step while using the routine 10 ppb standard. Calibration curves are linear in the 0.01 to 100 μg/L concentration range. The calibration points are: 0.01, 0.1, 0.5, 1, 5, 10, 20, 50, 100 μg/L. The correlation coefficient resulting from analysis is considered acceptable when r ^2^ ≥ 0.999 ( Figure 3).

SOP, standard operating procedure.

### Limit of detection (LOD) and limit of quantification (LOQ)

Limit of detection (LOD) is the smallest measure that can be detected with reasonable certainty for an analytical procedure, while the Limit of quantification (LOQ) is the smallest concentration that can be determined with accuracy and precision. There are several methods to calculate LOD and LOQ. We use the method based on the calibration curves of low concentration of target analyte ( Indrayanto, 2018).

LOD is expressed as:

LOD=3.3∗σ/S

and LOQ is expressed as:

LOQ=10∗σ/S

Where *σ* is the standard deviation of the regression and S is the slope of the calibration curve ( Al-Hakkani, 2019; Lister, 2005; Tietje & Brouder, 2010). The LOD and LOQ values obtained for our ICP-MS SOP are given in Table 3 and Table 4.

**Table 3.** LOD and LOQ for the ICP-MS SOP for the determination of biometals in hydrothermal fluids and sediments in the Giovannelli Lab. LOD, limit of detection; LOQ, limit of quantification; ICP-MS, inductively coupled plasma mass spectrometry; SOP, standard operating procedure.

	LOD		LOQ	
Analyte	μg/L	nM	μg/L	nM
^51^V	0.0227	0.446	0.0688	1.3514
^55^Mn	0.0231	0.4203	0.07	1.2736
^56^Fe	0.0318	0.569	0.0963	1.7242
^59^Co	0.0203	0.3436	0.0614	1.0413
^60^Ni	0.0365	0.6227	0.1108	1.887
^63^Cu	0.0207	0.3253	0.0626	0.9857
^75^As	0.0252	0.337	0.0765	1.0213
^78^Se	0.0322	0.408	0.0976	1.2364
^95^Mo	0.0158	0.1647	0.0479	0.4991
^111^Cd	0.0187	0.1666	0.0567	0.5047
^182^W	0.0157	0.0854	0.0476	0.2587

**Table 4.** LOD and LOQ for the ICP-MS SOP. LOD, limit of detection; LOQ, limit of quantification; ICP-MS, inductively coupled plasma mass spectrometry; SOP, standard operating procedure.

	LOD		LOQ	
Analyte	μg/L	nM	μg/L	nM
^47^Ti	0.054	1.12	0.162	3.39
^52^Cr	0.022	0.42	0.066	1.26
^71^Ga	0.020	0.29	0.061	0.87
^85^Rb	0.014	0.16	0.041	0.48
^88^Sr	0.016	0.18	0.047	0.54
^90^Zr	0.015	0.16	0.044	0.48
^93^Nb	0.007	0.07	0.021	0.22
^107^Ag	0.009	0.08	0.026	0.24
^133^Cs	0.013	0.10	0.041	0.31
^137^Ba	0.013	0.09	0.038	0.28
^181^Ta	0.013	0.07	0.040	0.22
^185^Re	0.015	0.08	0.047	0.25
^205^Tl	0.004	0.02	0.013	0.06
^208^Pb	0.016	0.08	0.047	0.23
^238^U	0.010	0.04	0.030	0.13

### Case study

**Figure 4.**
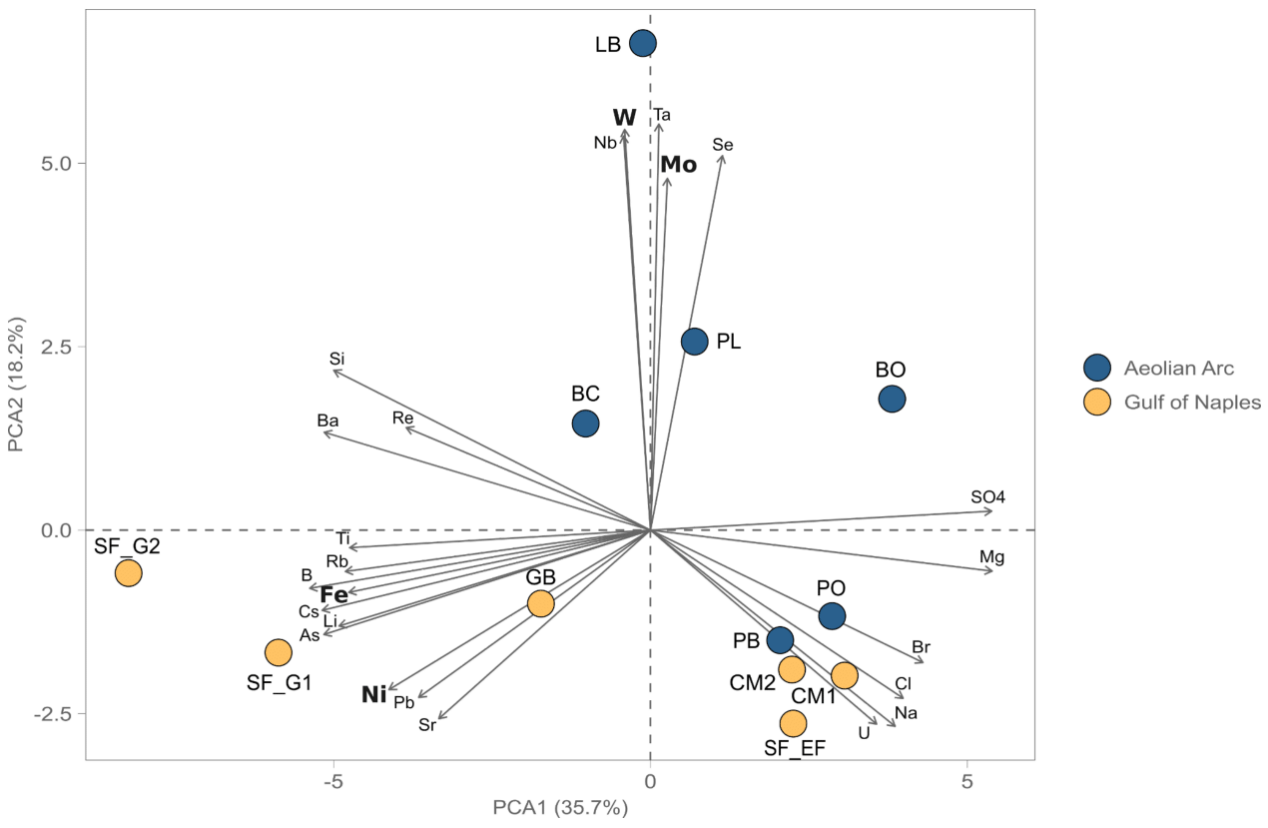
PCA analysis of hydrothermal fluids from the Aeolian Arc and the Gulf of Naples (Italy, unpublished data) based on trace metal concentration (trace metals with important biological functions are highlighted in bold). PCA, Principal component analysis.

The proposed procedure was applied to investigate the concentration of trace elements biologically significant ( *i.e.*, Fe, Co, Ni, Mo, Mn, W, As) in hydrothermal fluids collected on the AEO19 expedition to the islands of the Aeolian archipelago and in the Gulf of Naples during the FEAMP expedition. The data (number of determinations = 3) were used to conduct a principal component analysis (PCA) in order to cluster the samples based on the trace element profiles. As can be seen from Figure 4, the samples taken from the two active volcano islands of Vulcano and Panarea (BC, BO, PL and LB) are greatly influenced by higher concentrations of W and Mo. The samples from the Gulf of Naples and the samples from the inactive volcanic islands of the Aeolian Arquipelago cluster together on the lower half of the the PCA, with the high temperature sites of SF_G1, SF_G2 and GB that are strongly influenced by the concentrations of Ni and Fe. This suggests a differential mobility of the different trace metals in response to the diverse volcanic settings and the different temperatures of the investigated areas. Row data and corresponding precision are given in Table 5. The underlying data are available in GitHub ( Giovannelli, 2023b).

**Table 5.** Row data used for PCA analysis (expressed in ppb). Zeros represent values below detection limits.

SiteID	Li	Be	B	Al	Si	P	Ti	V	Cr	Fe	Co	Ni	Cu	Zn	As	Se	Rb	Sr	Zr	Nb	Mo	Ag	Cd	Cs	Ba	Ta	W	Re	Tl	Pb	U
LB	0.386 ± 0.027	0.001 ± 0.001	11.525 ± 0.092	0.425 ± 0.010	29.893 ± 1.746	0	0.003 ± 0.010	0	0	0	0	0	0.103 ± 0.002	0	0.151 ± 0.002	0.023 ± 0.021	0.696 ± 0.022	6.245 ± 0.076	0	0.05 ± 0.010	0.181 ± 0.013	0.001 ± 0.001	0	0.034 ± 0.003	0.115 ± 0.045	0.029 ± 0.002	0.177 ± 0.018	0.003 ± 0.001	0	0	0
PL	0.24 ± 0.018	0.002 ± 0.001	8.455 ± 0.214	0.358 ± 0.014	26.456 ± 1.253	0.342 ± 0.524	0	0.003 ± 0.001	0.014 ± 0.004	0	0.001 ± 0.001	0	0.06 ± 0.006	1.144 ± 0.087	0.15 ± 0.002	0.008 ± 0.009	0.223 ± 0.012	7.252 ± 0.114	0	0.02 ± 0.004	0.126 ± 0.003	0.004 ± 0.001	0	0.012 ± 0.002	0.052 ± 0.014	0.02 ± 0.001	0.065 ± 0.003	0.001 ± 0	0	0.003 ± 0	0.002 ± 0.001
BC	0.532 ± 0.026	0	7.623 ± 0.041	0.719 ± 0.009	23.413 ± 0.645	24.499 ± 0.514	0.012 ± 0.018	0.003 ± 0.001	0.024 ± 0.003	0	0	0	0.236 ± 0.002	2.797 ± 0.108	0.159 ± 0.004	0	0.38 ± 0.011	8.15 ± 0.047	0	0.014 ± 0.002	0.118 ± 0.002	0.002 ± 0.001	0.001 ± 0.001	0.095 ± 0.002	0.122 ± 0.006	0.019 ± 0.002	0.039 ± 0.002	0.001 ± 0.001	0.006 ± 0.001	0.004 ± 0.001	0.002 ± 0
BO	0.124 ± 0.013	0	1.329 ± 0.026	0.677 ± 0.011	9.16 ± 0.509	7.837 ± 0.417	0	0	0.019 ± 0.001	0	0	0	0.274 ± 0.002	0.746 ± 0.055	0.15 ± 0.001	0.016 ± 0.015	0.1 ± 0.003	1.443 ± 0.024	0.013 ± 0.003	0.011 ± 0.003	0.105 ± 0.004	0.002 ± 0.001	0.001 ± 0.001	0.024 ± 0.002	0.015 ± 0.020	0.018 ± 0.002	0.027 ± 0.001	0.001 ± 0	0	0	0
PO	0.224 ± 0.010	0	4.978 ± 0.051	0.007 ± 0.003	0.693 ± 0.557	0	0	0.001 ± 0.000	0	0	0	0	0.072 ± 0.005	0.118 ± 0.051	0.148 ± 0.002	0	0.146 ± 0.011	8.265 ± 0.061	0	0.005 ± 0.001	0.115 ± 0.001	0.001 ± 0.001	0	0.004 ± 0.001	0.001 ± 0.009	0.011 ± 0.002	0.011 ± 0.001	0	0.001 ± 0.001	0	0.003 ± 0.001
PB	0.25 ± 0.068	0	5.395 ± 1.023	0.372 ± 0.123	10.982 ± 9.247	0	0	0.001 ± 0.000	0	0	0	0	0.059 ± 0.004	0.764 ± 0.028	0.149 ± 0.002	0	0.138 ± 0.006	8.015 ± 0.056	0	0.004 ± 0.002	0.111 ± 0.001	0.007 ± 0.002	0.001 ± 0.001	0.003 ± 0.001	0.054 ± 0.004	0.01 ± 0.002	0.01 ± 0.002	0	0.001 ± 0.001	0.003 ± 0.001	0.003 ± 0
SF_G2	4.2 ± 0.081	0	33.273 ± 0.603	0.754 ± 0.021	35.889 ± 1.655	534.797 ± 11.878	0.056 ± 0.019	0.002 ± 0.001	0.022 ± 0.002	0.362 ± 0.018	0	0.057 ± 0.018	0.028 ± 0.003	1.637 ± 0.060	1.29 ± 0.033	0	1.869 ± 0.028	8.739 ± 0.097	0	0.011 ± 0.001	0.112 ± 0.003	0.006 ± 0.001	0	1.359 ± 0.019	0.162 ± 0.053	0.012 ± 0.002	0.024 ± 0.001	0.009 ± 0.001	0.001 ± 0.001	0.01 ± 0.001	0
CM2	0.186 ± 0.037	0	5.439 ± 0.065	0.096 ± 0.008	0	4.468 ± 0.353	0	0.002 ± 0.001	0	0	0	0.033 ± 0.015	0	0	0.151 ± 0.002	0	0.126 ± 0.009	8.03 ± 0.058	0	0.003 ± 0.001	0.111 ± 0.004	0.001 ± 0.001	0	0.001 ± 0.001	0	0.009 ± 0.002	0.007 ± 0.002	0	0	0.006 ± 0.001	0.003 ± 0.001
SF_G1	4.647 ± 0.015	0	36.822 ± 0.393	0.349 ± 0.010	40.46 ± 1.569	0	0.013 ± 0.027	0.001 ± 0.001	0.013 ± 0.006	0.078 ± 0.047	0	0.082 ± 0.038	0.011 ± 0.003	1.178 ± 0.133	1.541 ± 0.103	0	2.109 ± 0.110	9.717 ± 0.601	0	0.002 ± 0.002	0.101 ± 0.002	0.002 ± 0.001	0.001 ± 0.001	1.523 ± 0.080	0.204 ± 0.014	0.006 ± 0.002	0.015 ± 0	0	0.001 ± 0	0.009 ± 0.001	0
CM1	0.229 ± 0.042	0	5.551 ± 0.058	0	0	0	0	0.002 ± 0.002	0	0	0	0	0.015 ± 0.002	0	0.15 ± 0.001	0	0.121 ± 0.007	7.717 ± 0.084	0	0.001 ± 0	0.11 ± 0.003	0.002 ± 0.001	0	0.001 ± 0.001	0	0.006 ± 0.002	0.002 ± 0.001	0	0	0	0.003 ± 0.001
GB	4.777 ± 0.033	0	26.883 ± 0.324	0.103 ± 0.003	27.988 ± 0.848	0	0	0.001 ± 0.001	0	0.132 ± 0.098	0	0	0.117 ± 0.005	0	0.961 ± 0.117	0	2.482 ± 0.022	7.793 ± 0.055	0	0.001 ± 0.001	0.097 ± 0.001	0.002 ± 0	0.001 ± 0.001	0.148 ± 0.007	0.045 ± 0.011	0.005 ± 0.001	0.005 ± 0.001	0	0	0	0
SF_EF	0.242 ± 0.020	0	6.202 ± 0.112	0.699 ± 0.020	0	0	0	0.005 ± 0.001	0.007 ± 0.003	0	0	0.033 ± 0.025	0.018 ± 0.002	0.105 ± 0.041	0.153 ± 0.002	0	0.132 ± 0.012	7.74 ± 0.139	0	0.002 ± 0.001	0.111 ± 0.002	0.003 ± 0.001	0	0.006 ± 0.001	0.004 ± 0.021	0.005 ± 0.002	0.003 ± 0.001	0	0	0.009 ± 0.001	0.003 ± 0

## Ethics and consent statement

Ethical approval and consent were not required.

## Data Availability

Data available from: https://github.com/giovannellilab/GiovannelliLab_SOPs. The data for this manuscript are included in the folder ICP-MS_SOP. All the SOPs from the lab are released as a single repository to improve findability and reproducibility of the methods used in the lab. Archived data at the time of publication: https://doi.org/10.5281/zenodo.7614719 ( Giovannelli, 2023a). This project contains the following underlying data: Table1.csv. Contains the data for Table 1 of the manuscript. Table2.csv. Contains the data for Table 2 of the manuscript. Table3.csv. Contains the data for Table 3 of the manuscript. Table4.csv. Contains the data for Table 4 of the manuscript. Table5.csv. Contains the data for Table 5 of the manuscript. Data are available under the terms of the Creative Commons Attribution 4.0 International license (CC-BY 4.0).
