# Supplementary material for: Standard Operating Procedure for the determination of trace elements in hydrothermal fluids by Inductively Coupled Plasma Mass Spectrometry (ICP-MS)
Source: Open Res Eur. 2024 Jul 24;3:90. Originally published 2023 Jun 12. [Version 2] doi: 10.12688/openreseurope.15699.2 (PMC11316169; doi:10.12688/openreseurope.15699.2)
Supplement: Peer review history

# Reviewer report: Reviewer response for version 2

**DOI:** 10.21956/openreseurope.19125.r42437

**Published:** 2024-08-09

**Authors:** Maria Carmen Barciela-Alonso

**Affiliations:** 1 University of Santiago de Compostela, Coruña, Spain

The authors have made some changes taking into account the comments I have made. 

 1. Regarding LODs and LOQs, the authors should indicate in the text that the values presented in Tables 3 and 4 are instrumental limits of detection and quantification. 

 2. For the study of the accuracy of the method, the authors use the reference material ENV-META-12, for 11 elements. An alternative way to evaluate the accuracy of the method is to perform analytical recovery studies. The authors can use spiked samples.  That is, samples spiked with  known concentrations of the studied elements.  In my opinion the work could be completed by including analytical recovery studies.

Is the rationale for developing the new method (or application) clearly explained?

Yes

Is the description of the method technically sound?

Yes

Are the conclusions about the method and its performance adequately supported by the findings presented in the article?

Partly

If any results are presented, are all the source data underlying the results available to ensure full reproducibility?

Partly

Are sufficient details provided to allow replication of the method development and its use by others?

Partly

Reviewer Expertise:

Spectrometry, Trace element determination, envisonmental and biological samples, speciation

I confirm that I have read this submission and believe that I have an appropriate level of expertise to confirm that it is of an acceptable scientific standard.

# Reviewer report: Reviewer response for version 1

**DOI:** 10.21956/openreseurope.16961.r35307

**Published:** 2023-10-16

**Authors:** Zikri Arslan

**Affiliations:** 1 US Geological Survey, Sunrise Valley Drive Reston, USA

**Standard Operating Procedure for the analysis of trace elements in hydrothermal fluids by Inductively Coupled Plasma Mass Spectrometry (ICP-MS)**

 Introduction:

 "This Standard Operating Procedure (SOP) was developed in the Giovannelli Lab at the Department of Biology of the University of Naples Federico II and outlines the analysis of trace elements involved in biogeochemical cycles..."

 “Analysis of trace elements” is not correct terminology. Samples are analyzed for determination of .. So it should be determination of trace elements. Please correct in the title and entire manuscript.

 “Generally, microbial diversity is linked to environmental main physical-chemical parameters,”

 A bit confusing statement. Maybe better “ linked to a number of physical and chemical parameters, including, but not limited to,...”

 "Trace metals such as Fe, Co, Ni, Mo, W, V and Cu are used in specific sites of protein or enzymes, a set of microbial proteins containing redox-sensitive transition metals as their core catalytic but, despite the importance of this process, this relation has not been investigated in detail."

 This statement is not clear. Please revise and simplify..

 "Here we describe the SOP developed to routinely analyze such a wide range..."

 It’s better this way - 'Here we describe an SOP developed to routinely analyze such a wide range..'

**Methods**

 "ICP-MS is a technique that combines mass spectrometry to inductively coupled plasma, useful for determining trace metallic and non-metallic inorganic substances present in a sample. Liquid samples are taken through a peristaltic pump and nebulized, and the resulting aerosol is transported to the plasma torch (generally based on Argon gas) where the elements are ionized. ICP-MS can also measure major and minor elements not only trace elements. ICP-MS cannot measure substances. Please use “species” instead. The ions pass through a vacuum chamber where they are separated into photons, neutrons and interfering ions by an ion lens and a collision chamber (based on diverse inert or reactive gasses depending on the final application."

 What do mean by neutrons? Neutral species! I don’t think plasma produces neutrons!

 "Finally, each element passes through the detector to be counted."

 This not a correct statement. Elements do not pass through detector. Ions hit the detector. What passes through quadrupole are ions not elements. And separation is based on m/z ratio that occurs sequentially – called hopping mode. Please revise this section and describe corrected operation of quadruple mass filter and ion detection process.

 "We have developed this protocol using an Agilent ICP-MS 7900 (G8403A). The ICP-MS 7900 is a single quadrupole mass spectrometer that filters ions by mass to charge ratio (m/z). It consists of two pairs of rods connected to separate electrical supplies and the applied rod voltage can change rapidly, so the quadrupole can scan a wide mass range more than 10 times per second."

 Delete this section. Just provide instrument model and operating conditions.

 "Finally, there are many factors that can affect the final trace metal analysis results. Given the high sensitivity of the instrument (up to a few ppt for most metals), great care must be taken to minimize external contamination to the used equipment. To minimize possible contamination during field sampling and laboratory preparation procedures we always use specifically selected materials that have been treated to reduce the amount of contaminating metals (see below for details). For example, non-colored plastic tips and vials are preferred to colored ones, as the dye used for coloring the plastic can leach elements such as Cu, Fe, Zn and Cd, and all

 plastic and glass vials are acid washed to prevent leaching of Sb, Zn, Mn, Fe, Ba (Moody, 1983; Thomas, 2003). Indeed, it is preferable not to keep the samples in glass vials for a long period especially if the concentration of these elements are extremely low. The ICP-MS laboratory has restricted access to external personnel, and specific personal protective devices are always used to minimize common contamination from skin, hair, nails or jewelry."

 Please shorten this section and be concise. Provide recommended sample handling and storage conditions. It is already known that glass containers are not suitable for ICP-MS measurements.

 "Principle of functioning of ICP-MS"

 Please delete entire section. This is book knowledge. No need to describe operation principles of ICP-MS.

 "Standard Operating Practice in the Giovannelli lab. The method was developed using inductively coupled plasma mass spectrometry (ICP-MS)."

 Please provide model of ICP-MS instrument here again.

 "In order to reduce the dissolved solid, all samples are filtered in the field through a 0.22 μm filter and diluted prior to analysis."

 How do you reduce dissolved solids by filtration? Maybe more appropriate to say suspending particulate matter or colloidal matter. All dissolved species will pass through filter that can only trap particulate matter.

 "A certified reference material is used to verify the digestion efficiency."

 What sort of SRM was used for sediment QA/QC? Please provide list of used SRMs as well as recommended SRM. Also, indicate that SRM is used for verifying digestion efficiency and accuracy of ICP-MS analysis. Please provide purity grade for syringe filter and filter papers. What sort of pipettes are used? How did you check pipette calibrations?

 Cleaning procedure

 Please delete the first paragraph of this sections. “ICP-MS detects elements even in ppt …”

 Sample collection, preservation and storage

 "In the field, samples are collected and filtered through a 0.22 μm membrane filter in metal free plastic tubes and

 acidified with HNO3 2 %."

 Are samples acidified to 2% HNO3 or acidified with 2%HNO3? How much is used? Better to report final acidity of samples.

***Solid samples***

 "Before running ICP-MS, samples are microwaved digested following the EPA 3051A method for digestion of sediments, sludges, soils and oil. This digestion provides the total trace metal concentration present in the sample."

 Please revise as “Before running ICP-MS, samples are digested by microwave-assisted digestion system using the EPA 3051A method..”

 EPA 3051A with HNOe3-HCL is not a total digestion method. It is an extraction method. You won’t be able to digest silicates without HF. Please revise your statement.

 "2. After, weigh about 0.5 g using the analytical balance in a metal free container."

 Delete steps 5 through 7.

**Table 1.** **Results of the analysis of the microwave assessed acid digested reference materials.**

 There is only one SRM not SRMs. Please revise as “Recoveries for the target elements measured in ENV-META-12 certified referent material. Samples (n=xx) are digested according to EPA 3551A method."

 How many samples/replicates are digested? There should be error values in this table.

 Quality control (QC) of the analytical setup

 Figure 2: Why don’t you run QA-QC SRM during analysis. How do you check actual QA-QC in real samples. 10 ppb QC standard only verifies your calibration!

 Need to revise this run sequence and add QA-QC external SRMs after QC Standard. Also you need to verify calibration linearity and accuracy at the end of the analysis once unknowns are run.

 Analytical procedure

 Please delete this section. This is not analytical procedure but creating a method file that varies from instrument to instrument. Simply mention instrument preparation and daily performance test in this section etc.

 Instrument calibration

 "In our laboratory we routinely quantify biometal such as V, Mn, Fe, Co, Ni, Cu, As, Se, Mo, Cd and W"

 Never heard of biometal! Please use proper and simple terms – trace metals etc.

 Delete this section  - “Moreover, in the next future we will also quantify rare earth elements such as Ce, Dy, Er,

 Eu, Gd, Ho, La, Lu, Nd, Pr, Sm, Th, Tm, Y, Yb. The signal measured by ICP-MS is in count per second, in order to convert this into a concentration value, external calibration standards containing known concentration of elements are used to produce a calibration curve.”

 Provide how many standards are used and calibration range in this section..

**Table 2.** **LOD and LOQ for the ICP-MS SOP for the determination of biometals in hydrothermal fluids and sediments in**

**the Giovannelli Lab. **LOD, limit of detection; LOQ, limit of quantification; ICP-MS, inductively coupled plasma mass spectrometry; SOP, standard operating procedure.

 Values in Table 2 and 3 have too many significant figures. I think is acceptable to report LOD and LOQ for 2 significant figures. Like V  0.022 ug/L and 0.45 nM etc.

Is the rationale for developing the new method (or application) clearly explained?

Partly

Is the description of the method technically sound?

Yes

Are the conclusions about the method and its performance adequately supported by the findings presented in the article?

Yes

If any results are presented, are all the source data underlying the results available to ensure full reproducibility?

Yes

Are sufficient details provided to allow replication of the method development and its use by others?

Partly

Reviewer Expertise:

Analytical chemistry, ICP-MS method development, geological analysis

I confirm that I have read this submission and believe that I have an appropriate level of expertise to confirm that it is of an acceptable scientific standard, however I have significant reservations, as outlined above.

# Response

**Published:** 2024-02-15

**Authors:** Donato Giovannelli

**Affiliations:** Department of Biology, University of Naples Federico II, Naples, Italy

We want to thank the reviewer for the useful suggestions and comments. We have addressed all the questions raised and significantly improved the manuscript. We provide a point by point response below.

 1. We have changed as requested in the text and throughout the manuscript were appropriate. 

 2. We thank the reviewer for the comment, we have change the sentence.

 3. We thank the reviewer for the comment, we have simplify the sentence.

 4. We thank the reviewer for the comment, we have change the sentence.

 5. We wish to thank the reviewer for catching this typos.  We corrected them accordingly.

 6. Corrected.

 7. We have deleted this section and included a table (new Table.1 ) that highlights the performance feature of the instrument..

 8. We believe that a brief introduction to the concept of contamination and how to minimize these can be helpful for readers who are not familiar with these topics, and have added recommendations as suggested.

 9. We have minimize this section, but we think that is useful to give a brief introduction to the principle of functioning of the instrument.

 10. Corrected.

 11. We wish to thank the reviewer.  We corrected them accordingly.

 12. ENVIR-META-12 is a reference material recommended by the Italian Ministry for Environments directives (D.Lgs. 152 del 3 aprile 2006) for evaluating heavy metals in environmental matrices, and it is used for validation of the methods across laboratories in Italy. Syringe filters and paper are pretreated with nitric acid and consideration is given to the possible contamination by metals. Calibration of pipettes and instruments is carried out routinely following manufacturers specification.

 13. We believe that a brief section with the cleaning procedure can be useful to described the procedures that routinely adopt in the lab.

 14. Corrected.

 15. We agree with the reviewer with this sentence. We are interested in the metals available to microbes such as those related to carbonate. For this reason we don’t perform a total digestion up to silicate, but only partial digestion.

 16. We believe that this steps are important to describe the procedures that routinely adopt in the lab to increase reproducibility of the methods without leaving anything out of the description.

 17. Corrected. now Table 2

 18. We think that analyzing blank and a SRM (runs as a sample, not as a calibration point) every 10 samples is a good way to check the QA/QC, also for all samples and QA/QC SRM the %RDS is evaluated.

 19. The calibration linearity and accuracy are checked every analysis.

 20. We agree with the reviewer. We have change the title but we think that is advisable  to mention also the basic steps that are routinely carried out during the analysis.

 21. Biometals are subsets of metals that have important biological functions. For reference see Giovannelli et al., 2023 Nat Rev Earth and Env (https://doi.org/10.1038/s43017-023-00477-y), Hay Mele et al., 2023 Essay in Biochem (https://doi.org/10.1042/EBC20230012) and the great book by Crichton ¨Biological inorganic chemistry" (edited by Academic press).

 22. We think to give an indication that soon will be possible analyze also rare earth elements.

 23. We have add this section.

 24. Corrected.

 25. Corrected.

# Response

**Published:** 2024-04-24

**Authors:** Donato Giovannelli

**Affiliations:** Department of Biology, University of Naples Federico II, Naples, Italy

We want to thank the reviewer for the useful suggestions and comments. We have addressed all the questions raised and significantly improved the manuscript. We provide a point by point response below. "This Standard Operating Procedure (SOP) was developed in the Giovannelli Lab at the Department of Biology of the University of Naples Federico II and outlines the analysis of trace elements involved in biogeochemical cycles..." “Analysis of trace elements” is not correct terminology. Samples are analyzed for determination of .. So it should be determination of trace elements. Please correct in the title and entire manuscript. We have changed as requested in the text and throughout the manuscript were appropriate.  “Generally, microbial diversity is linked to environmental main physical-chemical parameters,” A bit confusing statement. Maybe better “ linked to a number of physical and chemical parameters, including, but not limited to,...” We thank the reviewer for the comment, we have change the sentence "Trace metals such as Fe, Co, Ni, Mo, W, V and Cu are used in specific sites of protein or enzymes, a set of microbial proteins containing redox-sensitive transition metals as their core catalytic but, despite the importance of this process, this relation has not been investigated in detail." This statement is not clear. Please revise and simplify.. We thank the reviewer for the comment, we have simplify the sentence. "Here we describe the SOP developed to routinely analyze such a wide range..." It’s better this way - 'Here we describe an SOP developed to routinely analyze such a wide range..' We thank the reviewer for the comment, we have change the sentence "ICP-MS is a technique that combines mass spectrometry to inductively coupled plasma, useful for determining trace metallic and non-metallic inorganic substances present in a sample. Liquid samples are taken through a peristaltic pump and nebulized, and the resulting aerosol is transported to the plasma torch (generally based on Argon gas) where the elements are ionized. ICP-MS can also measure major and minor elements not only trace elements. ICP-MS cannot measure substances. Please use “species” instead. The ions pass through a vacuum chamber where they are separated into photons, neutrons and interfering ions by an ion lens and a collision chamber (based on diverse inert or reactive gasses depending on the final application." What do mean by neutrons? Neutral species! I don’t think plasma produces neutrons! We wish to thank the reviewer.  We corrected them accordingly. "Finally, each element passes through the detector to be counted." This not a correct statement. Elements do not pass through detector. Ions hit the detector. What passes through quadrupole are ions not elements. And separation is based on m/z ratio that occurs sequentially – called hopping mode. Please revise this section and describe corrected operation of quadruple mass filter and ion detection process. Corrected We have developed this protocol using an Agilent ICP-MS 7900 (G8403A). The ICP-MS 7900 is a single quadrupole mass spectrometer that filters ions by mass to charge ratio (m/z). It consists of two pairs of rods connected to separate electrical supplies and the applied rod voltage can change rapidly, so the quadrupole can scan a wide mass range more than 10 times per second." Delete this section. Just provide instrument model and operating conditions. We have deleted this section and included a table (new Table.1 ) that highlights the performance feature of the instrument. "Finally, there are many factors that can affect the final trace metal analysis results. Given the high sensitivity of the instrument (up to a few ppt for most metals), great care must be taken to minimize external contamination to the used equipment. To minimize possible contamination during field sampling and laboratory preparation procedures we always use specifically selected materials that have been treated to reduce the amount of contaminating metals (see below for details). For example, non-colored plastic tips and vials are preferred to colored ones, as the dye used for coloring the plastic can leach elements such as Cu, Fe, Zn and Cd, and all plastic and glass vials are acid washed to prevent leaching of Sb, Zn, Mn, Fe, Ba (Moody, 1983; Thomas, 2003). Indeed, it is preferable not to keep the samples in glass vials for a long period especially if the concentration of these elements are extremely low. The ICP-MS laboratory has restricted access to external personnel, and specific personal protective devices are always used to minimize common contamination from skin, hair, nails or jewelry." Please shorten this section and be concise. Provide recommended sample handling and storage conditions. It is already known that glass containers are not suitable for ICP-MS measurements. We believe that a brief introduction to the concept of contamination and how to minimize these can be helpful for readers who are not familiar with these topics. "Principle of functioning of ICP-MS" Please delete entire section. This is book knowledge. No need to describe operation principles of ICP-MS. We have minimize this section, but we think that is useful to give a brief introduction to the principle of functioning of the instrument "Standard Operating Practice in the Giovannelli lab. The method was developed using inductively coupled plasma mass spectrometry (ICP-MS)." Please provide model of ICP-MS instrument here again. Corrected "In order to reduce the dissolved solid, all samples are filtered in the field through a 0.22 μm filter and diluted prior to analysis." How do you reduce dissolved solids by filtration? Maybe more appropriate to say suspending particulate matter or colloidal matter. All dissolved species will pass through filter that can only trap particulate matter. We wish to thank the reviewer.  We corrected them accordingly. "A certified reference material is used to verify the digestion efficiency." What sort of SRM was used for sediment QA/QC? Please provide list of used SRMs as well as recommended SRM. Also, indicate that SRM is used for verifying digestion efficiency and accuracy of ICP-MS analysis. Please provide purity grade for syringe filter and filter papers. What sort of pipettes are used? How did you check pipette calibrations? ENVIR-META-12 is a reference material recommended by the Italian Ministry for Environments directives (D.Lgs. 152 del 3 aprile 2006) for evaluating heavy metals in environmental matrices, and it is used for validation of the methods across laboratories in Italy. Syringe filters and paper are pretreated with nitric acid and consideration is given to the possible contamination by metals. Calibration of pipettes and instruments is carried out routinely following manufacturers specification. Cleaning procedure Please delete the first paragraph of this sections. “ICP-MS detects elements even in ppt …” We believe that a brief section with the cleaning procedure can be useful to described the procedures that routinely adopt in the lab Sample collection, preservation and storage "In the field, samples are collected and filtered through a 0.22 μm membrane filter in metal free plastic tubes and acidified with HNO3 2 %." Are samples acidified to 2% HNO3 or acidified with 2%HNO3? How much is used? Better to report final acidity of samples. Corrected Solid samples "Before running ICP-MS, samples are microwaved digested following the EPA 3051A method for digestion of sediments, sludges, soils and oil. This digestion provides the total trace metal concentration present in the sample." Please revise as “Before running ICP-MS, samples are digested by microwave-assisted digestion system using the EPA 3051A method..” EPA 3051A with HNOe3-HCL is not a total digestion method. It is an extraction method. You won’t be able to digest silicates without HF. Please revise your statement. We agree with the reviewer with this sentence. We are interested in the metals available to microbes such as those related to carbonate. For this reason we don’t perform a total digestion up to silicate, but only partial digestion. After, weigh about 0.5 g using the analytical balance in a metal free container." Delete steps 5 through 7. We believe that this steps are important to describe the procedures that routinely adopt in the lab to increase reproducibility of the methods without leaving anything out of the description Table 1. Results of the analysis of the microwave assessed acid digested reference materials. There is only one SRM not SRMs. Please revise as “Recoveries for the target elements measured in ENV-META-12 certified referent material. Samples (n=xx) are digested according to EPA 3551A method." How many samples/replicates are digested? There should be error values in this table. Corrected. now Table2 Quality control (QC) of the analytical setup Figure 2: Why don’t you run QA-QC SRM during analysis. How do you check actual QA-QC in real samples. 10 ppb QC standard only verifies your calibration! We think that analyzing blank and a SRM (runs as a sample, not as a calibration point) every 10 samples is a good way to check the QA/QC, also for all samples and QA/QC SRM the %RDS is evaluated Need to revise this run sequence and add QA-QC external SRMs after QC Standard. Also you need to verify calibration linearity and accuracy at the end of the analysis once unknowns are run. The calibration linearity and accuracy are checked every analysis Analytical procedure Please delete this section. This is not analytical procedure but creating a method file that varies from instrument to instrument. Simply mention instrument preparation and daily performance test in this section etc. We agree with the reviewer. We have change the title but we think that is advisable  to mention also the basic steps that are routinely carried out during the analysis  Instrument calibration "In our laboratory we routinely quantify biometal such as V, Mn, Fe, Co, Ni, Cu, As, Se, Mo, Cd and W" Never heard of biometal! Please use proper and simple terms – trace metals etc. Biometals are subsets of metals that have important biological functions. For reference see Giovannelli et al., 2023 Nat Rev Earth and Env (https://doi.org/10.1038/s43017-023-00477-y), Hay Mele et al., 2023 Essay in Biochem (https://doi.org/10.1042/EBC20230012) and the great book by Crichton ¨Biological inorganic chemistry" (edited by Academic press).  Delete this section  - “Moreover, in the next future we will also quantify rare earth elements such as Ce, Dy, Er, Eu, Gd, Ho, La, Lu, Nd, Pr, Sm, Th, Tm, Y, Yb. The signal measured by ICP-MS is in count per second, in order to convert this into a concentration value, external calibration standards containing known concentration of elements are used to produce a calibration curve.” we think to give an indication that soon will be possible analyze also rare earth elements Provide how many standards are used and calibration range in this section.. We have add this section  Table 2. LOD and LOQ for the ICP-MS SOP for the determination of biometals in hydrothermal fluids and sediments in the Giovannelli Lab. LOD, limit of detection; LOQ, limit of quantification; ICP-MS, inductively coupled plasma mass spectrometry; SOP, standard operating procedure. Values in Table 2 and 3 have too many significant figures. I think is acceptable to report LOD and LOQ for 2 significant figures. Like V  0.022 ug/L and 0.45 nM etc. Corrected

# Reviewer report: Reviewer response for version 1

**DOI:** 10.21956/openreseurope.16961.r35308

**Published:** 2023-10-16

**Authors:** Maria Carmen Barciela-Alonso

**Affiliations:** 1 University of Santiago de Compostela, Coruña, Spain

The manuscript describes a protocol for trace element analysis in sediments and hydrothermal fluids.  The topic is interesting and may be of great use to other researchers.  But in my opinion, the protocol needs to be improved to be applied.

 The aspects that should be revised are the following:

 In the section "Methods" on page 3, the first paragraph should be deleted. This information is published in the analytical instrumentation manuals. 

 In my opinion the section "Principle of functioning of ICP-MS" should be deleted. I would include only a table with the instrumental measurement conditions.

 In the section "Sample collection, preservation and storage" it should be indicated how the sediment sample is collected and how it is preserved.

 Regarding calibration, due to the complexity of the sample matrix, I think it would be appropriate to evaluate whether it is necessary to use the standard additions method.

 For the study of the accuracy of the method, the authors use the reference material ENV-META-12, for 11 elements. How do you evaluate the accuracy for the hydrothermal fluid and for the other elements in the sediment?

 I think there is an error in the LOD expression (LOD = 3.3∗ ­σ/ S). I think it is LOD = 3∗ σ / S.

 I do not understand the information in tables 2 and 3. It is the same detection limit for hydrothermal fluid and sediment?. In my opinion the LOD and LOQ should be given for the initial sample, considering the whole sample preparation procedure.

 The authors should include a table with the concentration values obtained for the samples.

Is the rationale for developing the new method (or application) clearly explained?

Yes

Is the description of the method technically sound?

Yes

Are the conclusions about the method and its performance adequately supported by the findings presented in the article?

Partly

If any results are presented, are all the source data underlying the results available to ensure full reproducibility?

Partly

Are sufficient details provided to allow replication of the method development and its use by others?

Partly

Reviewer Expertise:

Spectrometry, Trace element determination, envisonmental and biological samples, speciation

I confirm that I have read this submission and believe that I have an appropriate level of expertise to confirm that it is of an acceptable scientific standard, however I have significant reservations, as outlined above.

# Response

**Published:** 2024-02-15

**Authors:** Donato Giovannelli

**Affiliations:** Department of Biology, University of Naples Federico II, Naples, Italy

We want to thank the reviewer for the useful suggestions and comments. We have addressed all the questions raised and significantly improved the manuscript. We provide a point by point response below.

 1. We believe that a brief introduction to the instrumentation and working principle can be useful for non-expert readers, but we have omitted the interference section and added the citation as suggested.

 2. We have reduced this section and included a table (new Table.1 ) that highlights the performance feature of the instrument.

 3. We agree with the reviewer. We have added a brief clarification regarding how sediment are collected and preserved, which was not present in the initial draft

 4. We thank the reviewer for the comment, we will take into account the standard addition method. Currently we use internal standards added to the sample.

 5. We are seeking reference materials that contain other elements, and we have used this test material as it is the one recommended by the ministerial directives (D.Lgs. 152 del 3 aprile 2006) for evaluating heavy metals in environmental matrices.

 6. In literature we have found several ways to calculate limits of detection and quantification, and we added more references in the text. Using 3.3 instead of 3 is anyway more conservative.

 7. We have calculated the method LOD and LOQ  based on the standard calibration curves. Because of the significant variability in chemical and physical parameters, it's not feasible to use a single sample or a set of samples to determine detection and quantification limits that can be representative of all the samples we analyze. Therefore, we have chosen to assess the instrument's detection and quantification limits separately, while the limits for individual samples will be calculated during sample processing. Now Table 3 and Table 4.

 8. As in the previous statement, the detection limits are calculated during the analysis of the samples. Also we have added Table 5 with row data and precision of the same samples.

# Response

**Published:** 2024-04-24

**Authors:** Donato Giovannelli

**Affiliations:** Department of Biology, University of Naples Federico II, Naples, Italy

We want to thank the reviewer for the useful suggestions and comments. We have addressed all the questions raised and significantly improved the manuscript. We provide a point by point response below. In the section "Methods" on page 3, the first paragraph should be deleted. This information is published in the analytical instrumentation manuals.  We believe that a brief introduction to the instrumentation and working principle can be useful for non-expert readers, but we have reduced this section In my opinion the section "Principle of functioning of ICP-MS" should be deleted. I would include only a table with the instrumental measurement conditions. We have reduced this section and included a table (new Table.1 ) that highlights the performance feature of the instrument. In the section "Sample collection, preservation and storage" it should be indicated how the sediment sample is collected and how it is preserved. We agree with the reviewer. We have added a brief clarification regarding how sediment are collected and preserved, which was not present in the initial draft Regarding calibration, due to the complexity of the sample matrix, I think it would be appropriate to evaluate whether it is necessary to use the standard additions method. We thank the reviewer for the comment, we will take into account the standard addition method. Currently we use internal standards added to the sample. For the study of the accuracy of the method, the authors use the reference material ENV-META-12, for 11 elements. How do you evaluate the accuracy for the hydrothermal fluid and for the other elements in the sediment? We are seeking reference materials that contain other elements, and we have used this test material as it is the one recommended by the ministerial directives (D.Lgs. 152 del 3 aprile 2006) for evaluating heavy metals in environmental matrices I think there is an error in the LOD expression (LOD = 3.3∗ ­σ/ S). I think it is LOD = 3∗ σ / S. In literature we have found several ways to calculate limits of detection and quantification, and we added more references in the text. Using 3.3 instead of 3 is anyway more conservative. I do not understand the information in tables 2 and 3. It is the same detection limit for hydrothermal fluid and sediment?. In my opinion the LOD and LOQ should be given for the initial sample, considering the whole sample preparation procedure. We have calculated the method LOD and LOQ  based on the standard calibration curves. Because of the significant variability in chemical and physical parameters, it's not feasible to use a single sample or a set of samples to determine detection and quantification limits that can be representative of all the samples we analyze. Therefore, we have chosen to assess the instrument's detection and quantification limits separately, while the limits for individual samples will be calculated during sample processing. Now Table 3 and Table 4. The authors should include a table with the concentration values obtained for the samples. As in the previous statement, the detection limits are calculated during the analysis of the samples. Also we have added Table 5 with row data and precision of the same samples.

# Reviewer report: Reviewer response for version 1

**DOI:** 10.21956/openreseurope.16961.r34850

**Published:** 2023-09-27

**Authors:** Sadhan Bijoy Deb

**Affiliations:** 1 RACD, Bhabha Atomic Research Centre Medical Division, Mumbai, Maharashtra, India

This manuscript depicts the development and establishment of a SOP for quantification of trace elements in hydrothermal fluids and sediments by ICP-QMS. The trace metals includes both geological and biological relevant elements. The SOP was applied to analysis of real samples collected on the AE019 expedition to the islands of Aeolian archipelago and in the gulf of Naples during the FEAMP expedition. The sediments were digested by Microwave Digestion and the liquid samples were diluted prior to analysis. The obtained data was employed to construct a PCA of trace metal concentration corresponding to the regions considered.

 The data has the potential to be used in future for investigating the role of trace elements on microbial functional diversity control. Microorganisms play a crucial role in biogeochemical cycle that in turn is responsible for controlling the interaction between geosphere and biosphere.

**Comments**

1. Under the heading “Methods (pg3)”, the instrumentation and working principle (including interferences) of ICP-QMS are explicitly described in the literature. The authors can omit these details from the manuscript and cite the appropriate references. This would reduce the repeat and that the pertinent focus of the present work can blossom out.
2. Under the heading “Methods (pg3)”, the authors had mentioned that “it is advisable to dilute the sample, to keep total dissolved solids (TDS) below 0.2%”. Appropriate reference(s) has to be cited in support of the statement. In the same paragraph, “For comparison with a 50% dilution of seawater (1.5% NaCl final concentration) there is no significant cone blockage and signal stability remains good.” Again reference has to be cited if this sentence is retained.
  The above statements are contradictory to each other. The authors should clearly state the level of dilution they followed in their work and the corresponding TDS achieved in the final solution. The authors can remove the contradicting sentences.
3. The information given under the heading “Principle of functioning of ICP-MS (Pg 4)”, are already available in literature. Hence, I would recommend the removal of text which is available in literature and the authors should cite the relevant references.
  The performance features of the instrument (power, gas flow rate, etc.) should be mentioned in a tabular format and not described in words.
4. There is repeat of same text (cleaning, sample collection, etc.) under the headings – “Standard operating practice in the Giovannelli lab (pg4)”, “cleaning procedure (pg 6)”, and “sample collection, preservation and storage (pg 6)”. The authors should avoid such repetition in the manuscript. 
5. Three biologically relevant elements i.e., Fe, Mo & W are absent in certified test material ENV-META-12. The authors should explore other CRMs of sludge/sediment which contains these elements, so that the digestion efficiency of these elements are verified.
6. The accuracy of the SOP for analysis of hydrothermal liquid samples has not been mentioned. The methodology can be validated by using a CRM or standard addition technique.
7. The relevant references for LOD and LOQ had been provided in the text. Hence the definitions of LOD & LOQ need not be given. 
8. The raw data [with corresponding precision and number of determinations (N)] prior to PCA analysis should be provided as supporting information

Is the rationale for developing the new method (or application) clearly explained?

Yes

Is the description of the method technically sound?

Yes

Are the conclusions about the method and its performance adequately supported by the findings presented in the article?

Yes

If any results are presented, are all the source data underlying the results available to ensure full reproducibility?

Partly

Are sufficient details provided to allow replication of the method development and its use by others?

Yes

Reviewer Expertise:

Trace metallic analysis, by ICP-MS, in nuclear, geological and environmental samples. Microwave digestion of refractory materials. Gravimetric quantification of major elements in a matrix. Minor element anaysis by UV-Vis spectroscopy

I confirm that I have read this submission and believe that I have an appropriate level of expertise to confirm that it is of an acceptable scientific standard, however I have significant reservations, as outlined above.

# Response

**Published:** 2024-02-15

**Authors:** Donato Giovannelli

**Affiliations:** Department of Biology, University of Naples Federico II, Naples, Italy

We want to thank the reviewer for the useful suggestions and comments. We have addressed all the questions raised and significantly improved the manuscript. We provide a point by point response below.  

 1. We believe that a brief introduction to the instrumentation and working principle can be useful for non-expert readers, but we have omitted the interference section and added the citation as suggested.

 2. Thank you for pointing this out! We agree and have removed these confusing sentences.

 3. We significantly reduced the text and included a table (new Table.1 ) that highlighted the performance feature of the instrument, as indicated by the reviewer.

 4. In the section Standard operating practice we have removed the repetition, present in the section below.

 5. Iron, molybdenum, and tungsten are transition metals included among the metals we have examined. We do not anticipate significant deviations in their recoveries. We are seeking reference materials that contain these elements, and we have used this test material as it is the one recommended by the ministerial directives (D.Lgs. 152 del 3 aprile 2006) for evaluating heavy metals in environmental matrices.

 6. We have evaluated the accuracy and recovery using the internal standard addition and QC standard for each sample set.

 7. As not everyone might be familiar with this concept, we believe that a brief introduction to the concept of limits of detection and quantification can be helpful.

 8. We have added the number of determination and a table with row data and precision (Table 5) as requested.

# Response

**Published:** 2024-04-24

**Authors:** Donato Giovannelli

**Affiliations:** Department of Biology, University of Naples Federico II, Naples, Italy

We want to thank the reviewer for the useful suggestions and comments. We have addressed all the questions raised and significantly improved the manuscript. We provide a point by point response below.

 Under the heading “Methods (pg3)”, the instrumentation and working principle (including interferences) of ICP-QMS are explicitly described in the literature. The authors can omit these details from the manuscript and cite the appropriate references. This would reduce the repeat and that the pertinent focus of the present work can blossom out. We believe that a brief introduction to the instrumentation and working principle can be useful for non-expert readers, but we have omitted the interference section and added the citation as suggested. Under the heading “Methods (pg3)”, the authors had mentioned that “it is advisable to dilute the sample, to keep total dissolved solids (TDS) below 0.2%”. Appropriate reference(s) has to be cited in support of the statement. In the same paragraph, “For comparison with a 50% dilution of seawater (1.5% NaCl final concentration) there is no significant cone blockage and signal stability remains good.” Again reference has to be cited if this sentence is retained.The above statements are contradictory to each other. The authors should clearly state the level of dilution they followed in their work and the corresponding TDS achieved in the final solution. The authors can remove the contradicting sentences. Thank you for pointing this out! We agree and have removed these confusing sentences. The information given under the heading “Principle of functioning of ICP-MS (Pg 4)”, are already available in literature. Hence, I would recommend the removal of text which is available in literature and the authors should cite the relevant references. The performance features of the instrument (power, gas flow rate, etc.) should be mentioned in a tabular format and not described in words. We significantly reduced the text and included a table (new Table.1 ) that highlighted the performance feature of the instrument, as indicated by the reviewer. There is repeat of same text (cleaning, sample collection, etc.) under the headings – “Standard operating practice in the Giovannelli lab (pg4)”, “cleaning procedure (pg 6)”, and “sample collection, preservation and storage (pg 6)”. The authors should avoid such repetition in the manuscript.  In the section Standard operating practice we have removed the repetition, present in the section below Three biologically relevant elements i.e., Fe, Mo & W are absent in certified test material ENV-META-12. The authors should explore other CRMs of sludge/sediment which contains these elements, so that the digestion efficiency of these elements are verified. Iron, molybdenum, and tungsten are transition metals included among the metals we have examined. We do not anticipate significant deviations in their recoveries. We are seeking reference materials that contain these elements, and we have used this test material as it is the one recommended by the ministerial directives (D.Lgs. 152 del 3 aprile 2006) for evaluating heavy metals in environmental matrices. The accuracy of the SOP for analysis of hydrothermal liquid samples has not been mentioned. The methodology can be validated by using a CRM or standard addition technique. We have evaluated the accuracy and recovery using the internal standard addition and QC standard for each sample set. The relevant references for LOD and LOQ had been provided in the text. Hence the definitions of LOD & LOQ need not be given.  We believe that a brief introduction to the concept of limits of detection and quantification can be helpful for readers who are not familiar with these topics. The raw data [with corresponding precision and number of determinations (N)] prior to PCA analysis should be provided as supporting information We have added the number of determination and a table with row data and precision (Table5)
